# Macrodystrophia Lipomatosa: A Case Report and Relevant Anatomical Considerations

**DOI:** 10.5334/jbsr.3522

**Published:** 2024-03-26

**Authors:** Inês Da Mata, António Proença Caetano

**Affiliations:** 1 Department of Radiology, Unidade Local de Saúde São José (ULS São José), Lisbon, Portugal; 2 Interventional Radiology Unit, Hospital Curry Cabral - Unidade Local de Saúde São José (ULS São José), Lisbon, Portugal; Nova Medical School, Universidade NOVA de Lisboa, Lisbon, Portugal

**Keywords:** Macrodactyly, macrodystrophia lipomatosa, musculoskeletal, MRI

## Abstract

Macrodactyly, a often congenital anomaly, entails abnormal enlargement of digits, predominantly affecting hands or feet, either in isolation or as part of a syndromic condition. The authors present a case of Macrodystrophia Lipomatosa (ML), a form of macrodactyly, in a 62-year-old patient, emphasizing macrodactyly manifestations through clinical and radiological assessments. Additionally, the authors explore anatomical aspects related to nerve distribution in affected digits, providing a comprehensive understanding of ML.

*Teaching Point:* Explore macrodactyly, emphasizing digits nerve plexus anatomy, which can reveal crucial clues for diagnosing a specific form of this anomaly.

## Introduction

Macrodactyly is a rare musculoskeletal anomaly, often congenital, characterized by abnormal enlargement of one or more digits that can occur either as an isolated finding or as part of a syndromic association [1]. Pathogenesis is still not fully understood, and various hypotheses have been proposed, including abnormal regulation of growth factors or genetic abnormalities such as “gain-of-function” mutations, which activate the *protein kinase* PI3K/AKT cell signaling pathway [2]. The affected digit(s) can show enlargement in various types of mesenchymal tissue, including muscle, bone, nerves, and fibro-adipose tissue.

## Case Report

The authors present a case of a 62-year-old female patient with a 10-year history of progressive enlargement of the third digit of the left hand presenting to the orthopedic surgeon appointment. The patient did not recall any significant trauma or other medical conditions related to the development of the finger deformity. She reported no significant pain or functional limitations.

**Figure 1:**
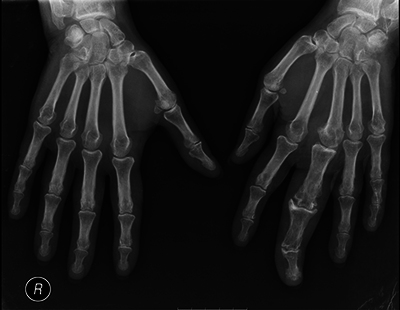
Hand radiograph, anterior–posterior projection, showing soft tissue swelling and skeletal hypertrophy affecting the 3rd digit of the left hand.

**Figure 2:**
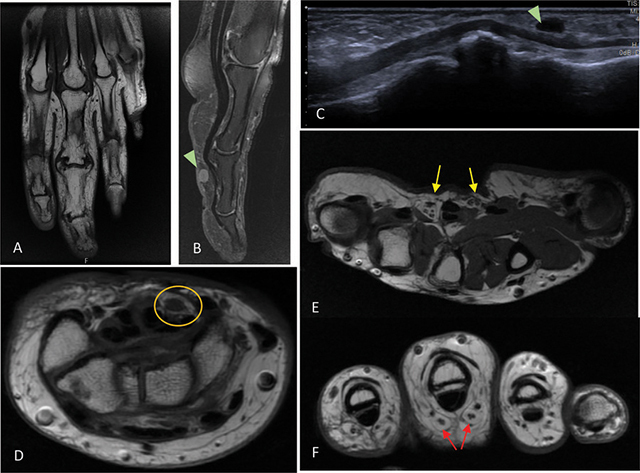
Hand MRI. **(A–B)** Coronal T1-weighted and sagittal PDFS images showing lipomatous hypertrophy of the subcutaneous tissue of the third finger, predominant on the volar side. Small synovial cysts are present (Fig. 2B) and coincide with anechoic structures on ultrasound (Fig. 2C). **(D)** Axial T1-weighted image with no significant abnormalities of the median nerve (orange circle) and no space-occupying lesions. Axial T1-weighted images through the metacarpal heads **(E)** and proximal phalanx **(F)** show subtle fatty infiltration of the common volar digital nerves (yellow arrow) and proper volar digital nerves (red arrows). PD—proton density. FS—fat-saturated.

Radiographic examination of the hands revealed volumetric enlargement of the proximal, middle, and distal phalanges of the third digit of the left hand and adjacent soft tissue swelling. Additionally, degenerative changes were observed in the interphalangeal and metacarpophalangeal joints, including marginal osteophytes, subchondral sclerosis, and joint space reduction, most prominent in the proximal and distal interphalangeal joints (Figure 1). Further investigation included magnetic resonance imaging (MRI) of the left hand and wrist (Figure 2).

Based on the clinical presentation, radiographic, and MRI findings, a diagnosis of *macrodystrophia lipomatosa* (ML) was assumed. The patient was informed about the available treatment options, including surgical intervention, but declined to undergo surgery.

## Discussion

*ML* is a rare, often unilateral, congenital non-hereditary form of macrodactyly and involves one or multiple digits of the extremities. Pathological examinations reveal predominant fatty infiltration and hypertrophy of subcutaneous tissue, nerve sheaths, and periosteum, leading to localized bone overgrowth [1]. Lipofibromatous hamartoma of the nerves is frequently associated with *ML*, with the median nerve being most commonly affected [3]. Additionally, secondary degenerative and deforming bone changes are common features of macrodactyly, which manifest with new bone formation and bony spurs.

Management strategies for ML may vary depending on the severity of symptoms, functional impairment, and patient choice. Conservative options such as orthotics can be considered initially, and surgical interventions, including debulking procedures, osteotomy, and tendon transfer, may be necessary in cases of significant functional limitation or cosmetic concerns [1, 2]. Long-term follow-up and further research are necessary to monitor the progression of the condition and assess the impact on hand function and quality of life.

### Anatomical Considerations

The hand and fingers receive sensory and motor innervation through the nerve plexus originating from the median, radial, and ulnar nerves (schematic drawings 3 and 4). After crossing the carpal tunnel, the median nerve divides into lateral and medial branches, with the former originating a motor recurrent branch for the thenar eminence muscles and three proper volar digital nerves for the thumb and radial aspect of the second finger [4]. The medial branch originates cutaneous sensory common volar digital nerves, each further branching into two proper volar digital nerves for the second and third fingers and the radial side of the fourth finger [4]. The ulnar nerve, post-Guyon’s canal tunnel, divides into one or two superficial sensory branches for the ulnar side of the fourth and fifth fingers, and a deep branch for the hypothenar eminence muscles [4]. Additionally, the superficial dorsal branch of the radial nerve contributes to sensation of the radial aspect of the dorsal hand, dorsal thumb, second finger, third finger, and radial half of the fourth finger proximal to the distal interphalangeal joint [4].

**Figure 3:**
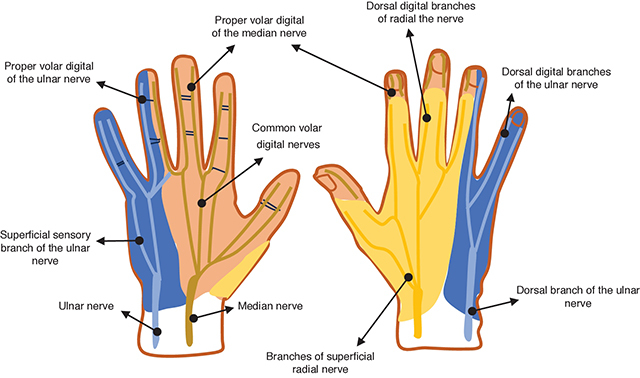
Sensorial cutaneous innervation of the hand and fingers (right: volar view and left: dorsal view).

**Figure 4:**
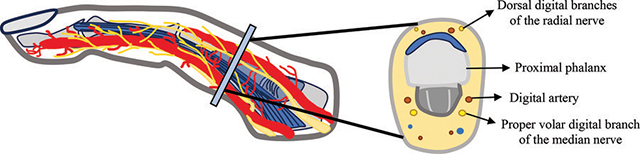
Digit’s relevant anatomical structures (axial view of the proximal phalanx).

## Conclusion

The authors present a case of true macrodactyly of one digit, which typically presents with digital nerve territory involvement. A detailed understanding of the anatomy of the hand and fingers, especially the digital nerve plexus, is crucial to facilitate the detection of common abnormalities associated with macrodactyly.
